# Supplementary material for: A CRISPRi library screen in group B Streptococcus identifies surface immunogenic protein (Sip) as a mediator of multiple host interactions
Source: Infect Immun. 2025 Mar 21;93(4):e00573-24. doi: 10.1128/iai.00573-24 (PMC11977309; doi:10.1128/iai.00573-24)
Supplement: Supplemental material — Fig. S1; Supplemental captions. [file iai.00573-24-s0002.docx]

**Supplemental Data 1 Caption:**

**Tab 1 (CNCTC Signal Peptide Genes)** Data table listing the set of CNCTC 10/84 signal peptide-containing genes that were also present among a subset of the 654-genome screening collection. The column labeled “Conserved” indicates those 66 genes that were in the CRISPRi library. **Tab 2 (Conserved Knockdown Library)** Lists the knockdown strains in the CRISPRi library. The protospacer number in the “Gene-Protospacer ID” column indicates where in the gene coding sequence dCas9 was targeted for that knockdown strain. **Tab 3** (**RT-qPCR)** shows normalized expression data from the CRISPRi library strains. **Tab 4** **(CRISPRi Cytokine Profiling)** shows ELISA results from THP-1 macrophage coincubation with ethanol-killed strains from the CRISPRi library.

**
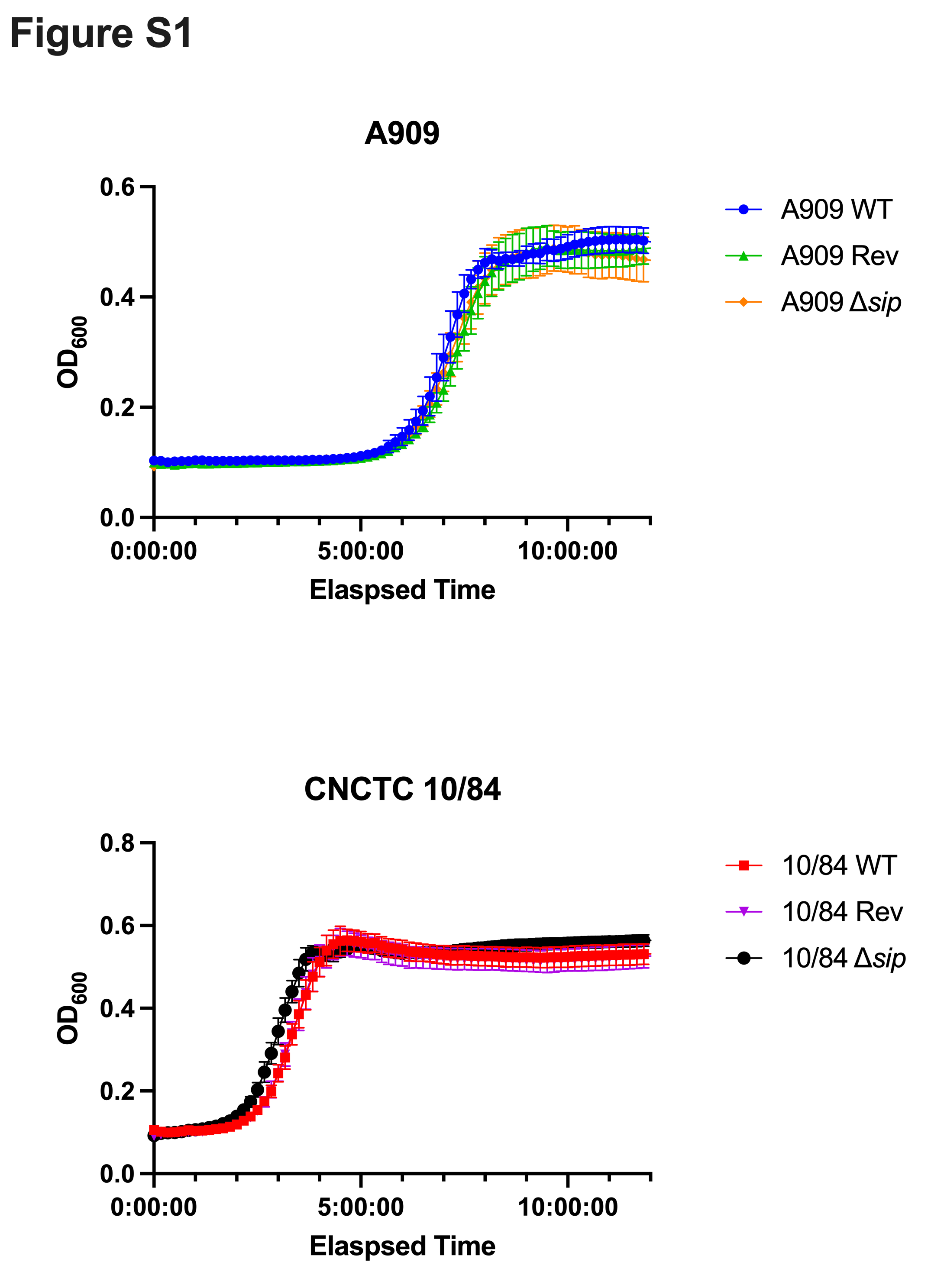
**

**Supplemental Figure S1: No growth defect *in vitro* due to *sip* mutation.** Wild type (WT), Δ*sip*, and *rev* strains—in A909 and CNCTC 10/84 backgrounds—were grown in independent biological triplicate samples at 37°C with OD_600_ measurements recorded every 10 minutes.
